# Semi-quantitative CT severity scoring as a predictor of development of post-COVID syndrome

**DOI:** 10.1186/s43055-021-00483-4

**Published:** 2021-04-13

**Authors:** Mohammad A. Saad, Ahmed F. El Khateeb, Mona I. Ahmed, Ahmed M. Magdy

**Affiliations:** 1 Radiology Department, Faculty of Medicine, Fayoum University, Fayoum, Egypt; 2 Department of Intensive Care Unit, Faculty of Medicine, Fayoum University, Fayoum, Egypt; 3 Department of Chest Disease and Tuberculosis, Faculty of Medicine, Fayoum University, Fayoum, Egypt

**Keywords:** COVID-19 pneumonia, Post-COVID syndrome, CT chest, CT severity scoring

## Abstract

**Background:**

Following COVID-19 pandemic, clinical description focused on the clinical presentation of patients in the acute stage of the disease. More recently, data have emerged that some patients continue to experience symptoms related to COVID-19 after the acute phase of infection has subsided (post-COVID syndrome). Although characteristics of post-COVID syndrome have been well described, less is known about the possible invitations during acute illnesses that can predict such syndrome. Our study is a prospective study aiming at assessment of CT severity scoring in the acute phase of COVID-19 pneumonia as a predictor for development of post-COVID syndrome in recovering patients.

**Results:**

A total of 192 symptomatic COVID-19 patients between April 2020 and October 2020 were enrolled in this single-center study, and high-resolution chest CT examinations were evaluated for CT severity scoring. Data were matched with the long-term clinical outcome. CT severity score was significantly higher in patients who developed post-COVID symptoms (*p* < 0.001). A CT score of > 7 was associated with an increased risk and was found to be predictive of condition development with sensitivity (95.9%), specificity (96%), positive predictive value (95.92%), negative predictive value (96%), and accuracy (95.96%).

**Conclusions:**

CT severity scoring can help in predicting the long-term outcome of COVID-19 patients with cutoff value of CT-SSS > 7 showing highest sensitivity and specificity for predicting development of post-COVID syndrome.

## Background

Severe acute respiratory syndrome coronavirus disease 2019 (COVID-19) was firstly described in Wuhan, China, during December 2019 in a series of 41 patients presenting with unspecified forms of pneumonias [1].

Following COVID-19 pandemic, clinical description focused on the clinical presentations of patients in the acute stage of the disease. More recently, data have emerged that some patients continue to experience symptoms related to COVID-19 after the acute phase of infection has subsided. Most patients who have coronavirus disease 2019 (COVID-19) will recover completely within a few weeks. But some people continue to experience symptoms after their initial recovery. The most common signs and symptoms may include [2]:

- Easy fatigability
- Dyspnea
- Cough
- Arthralgia
- Chest pain

Other long-term signs and symptoms may include:

- Muscle pain or headache
- Tachycardia
- Loss of smell or taste
- Memory, concentration, or sleep problems
- Rash or hair loss

High-resolution chest CT has 97% sensitivity for the diagnosis of COVID-19 pneumonia after a mean interval of 5 days [3]. The typical chest CT findings in COVID-19 pneumonia are bilateral, peripheral, and basal predominant ground-glass opacities (GGOs) with or without consolidation and broncho-vascular thickening [4]. In addition, atypical chest CT findings include central upper lobe predominance, masses, nodules, cavitations, tree-in-bud sign, lymphadenopathy, and pleural effusion [5].

Chest computed tomography severity score was proposed by Yang et al. and Pan et al. and was published in *Radiology* in 2020 [6, 7]. It was created to help assess COVID-19 effect on the initial scan obtained at admission and provides an objective approach to identify patients in need of admission to hospital. The score (CT-SSS) is an adaptation of a method previously used during the SARS epidemic of 2005 [8].

This scale uses lung opacification as an equivalent for extension of the disease in the lungs.

We report our experience on a cohort of symptomatic patients who underwent high-resolution chest CT following chest clinic and emergency room clinical triage.

Our hypothesis was that CT severity scoring system during the acute phase of the disease would allow recognition of those patients who are more likely to develop post-COVID syndrome on the long-term follow-up.

This study aimed to highlight the role of CT severity scoring for predicting the long-term outcome of COVID-19 patients.

## Methods

### Patients

The present study was a single-center prospective analysis conducted on a total of 192 symptomatic patients referred from the chest clinic and emergency department in our hospital between April 2020 and October 2020. The patients underwent non-contrast high-resolution multi-slice CT scan of the chest done between 4 and 10 days from onset of symptoms in the Radiology Department in our hospital, and followed up, thereafter for 2–3 months in the chest outpatient clinic. The local ethical committee approved this prospective study and written informed consent was taken.

#### Inclusion criteria

Patients with the suspicion of COVID-19 pneumonia who showed positive results of RT-PCR and positive CT findings of COVID pneumonia.

#### Exclusion criteria

Patients less than 18 years old, patients who died in the acute disease, inadequate follow-up.

### Methods

All patients were subjected to:

- Full history taking.
- Clinical scoring for disease severity was established following the criteria provided by the Chinese Center of Disease Control (CDC) [9]: mild disease including non-pneumonia or mild pneumonia (mild symptoms without dyspnea; respiratory frequency < 30/min; blood oxygen saturation (SpO_2_) > 93%); severe disease including dyspnea (respiratory frequency ≥ 30/min, SpO_2_ ≤ 93%); and critical disease including adult respiratory distress syndrome (ARDS) or respiratory failure, septic shock, and/or multiple organ dysfunction (MOD) or failure (MOF).
- CT chest: Single CT scanner (Toshiba Aquilion Prime 160; Toshiba medical systems, Japan) was used for examining all patients. Acquisition parameters were as follows: 120 kVp, 100–180 mAs, pitch 0.75–1.5, and collimation 0.625. Images were reconstructed with a 1-mm slice thickness in all cases using the classic filtered back-projection method with all volumetric chest CT reviewed at lung window of 1500 WW and − 500 WL and mediastinal window of 400 WW and 60 WL. Coronal and sagittal multi-planar reconstructions were evaluated for better assessment of the extent of the disease. Color-coded images were reviewed, with the Dicom viewer automatically analyzing lung CT density distribution into different colors by entering the density range and selecting the desired color, which corresponds to the area that the density range occupies in the image. Normally aerated lung density range (−750 to − 950 HU) and the value range of GGO (− 749 HU to − 300 HU) and for consolidation (− 299 HU to 50 HU) [10].
- All possible infection control measures were arranged in CT cases, consisting of prompt sanitation of CT facility and patient’s isolation. Patients were examined in the supine position during full inspiration and breathe hold starting from the level of thoracic inlet to the upper pole of the right kidney.
- Image analysis: The studies were reviewed on PACS system (Paxera Ultima version 6.0.0.1). Two chest radiologists (with 13 years and 25 years of experience in interpreting chest CT images) independently evaluated all patients, blinded to clinical characteristics and laboratory data. Any disagreement between the two observers was resolved by consensus. Each of the following lung parenchymal findings was evaluated: GGO, consolidation, crazy paving, vacuolar sign, pulmonary nodules, lobar pneumonia, and lung cavitation as well as associated traction bronchiectasis and vascular thickening. The involved lung lobes and lesions’ pattern of distribution whether peripheral, peripheral and central, patchy, or diffuse were assessed. Also fibrosis, sub-pleural bands, reversed “halo sign,” pleural effusion, and lymphadenopathy were also mentioned. CT findings were categorized according to one of the CT severity scoring system (CT-SSS) categories. In all cases, a semi-quantitative CT severity scoring proposed by Yang et al. and Pan et al. [6, 7] was calculated for each of the 5 lobes regarding the extent of pathologic involvement, as follows: 0, no involvement; 1, < 5% involvement; 2, 5–25% involvement; 3, 26–50% involvement; 4, 51–75% involvement; and 5, > 75% involvement. The resulting global CT score was the sum of each individual lobar score from 0 to 25.

Following initial recovery, all patients were followed in the chest outpatient clinic of our hospital at 7, 30, and 60 days. Some cases were followed for additional 4th visit after 90 days. Persisting symptoms were defined as the presence of abnormal symptoms or signs after recovery from the acute phase of COVID pneumonia and persistence of these symptoms for the next 2–3 months; such symptoms are not explained by an alternative diagnosis. The following are the signs and symptoms suggesting post-COVID syndrome: “weight loss ≥ 5%, dyspnea or asthenia, chest pain, palpitations, anosmia, headache, cutaneous signs, arthralgia, myalgia, digestive disorders, or fever.”

### Statistical analysis

Data were coded and entered using the statistical package SPSS (Statistical Package for the Social Sciences) version 26 (IBM Corp., Armonk, NY, USA). Data was summarized using mean, standard deviation, median, and minimum and maximum in quantitative data and using frequency (count) and relative frequency (percentage) for categorical data. Standard diagnostic indices including sensitivity, specificity, positive predictive value (PPV), negative predictive value (NPV), and diagnostic efficacy were calculated [11]. ROC curve was constructed with area under curve analysis performed to detect best cutoff value of CT-SSS for development of post-COVID syndrome. *P* value less than 0.05 was considered as statistically significant.

## Results

Starting from April 2020 to October 2020, a total of 192 patients referred from the chest clinic and emergency department in our institution with clinical suspicion of COVID-19 pneumonia, who showed positive RT-PCR results, underwent a non-contrast, high-resolution multi-slice CT scan of the chest done between 4 and 10 days from onset of symptoms.

From the original cohort of 208 cases with the suspicion of COVID-19 infection and a positive RT-PCR test for COVID-19, 16 patients died in the acute stage and were excluded from this study with the final population including 192 patients (127 males, 65 females; mean age was 37.86±11.8 years).

Regarding the presence of comorbidities, the percent of cases with at least one medical comorbidity (diabetes, hypertension, chronic chest, or heart diseases) was 27.1%.

Regarding the clinical disease severity of COVID19 in our patients, mild cases were 163 (85.0%), severe cases 17 (8.6%), and critical cases 12 (6.4%) (Table 1).

**Table 1:** COVID-19 clinical severity distribution

		Count	%
**COVID-19 clinical severity distribution**	**Mild**	163	85.0
	**Severe**	17	8.6
	**Critical**	12	6.4

**Fig. 1:**
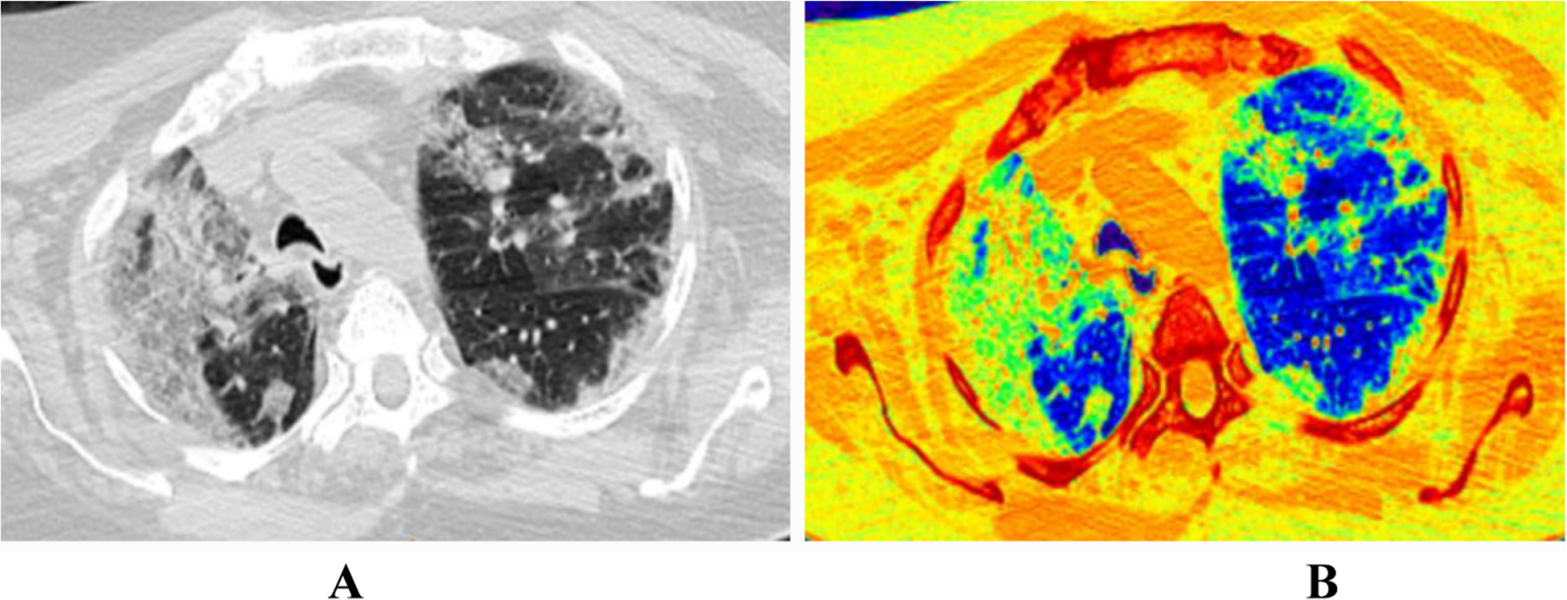
A 55-year-old female patient with first presentation with dyspnea and cough; CT-SSS was 14. Follow-up revealed development of post-COVID syndrome in the form of persistent cough after 60 days from the onset. **a** MDCT chest, axial cuts show widespread bilateral predominantly peripheral patchy and confluent ground glass opacities with septal and vascular thickening. **b** Corresponding color coded images

**Fig. 2:**
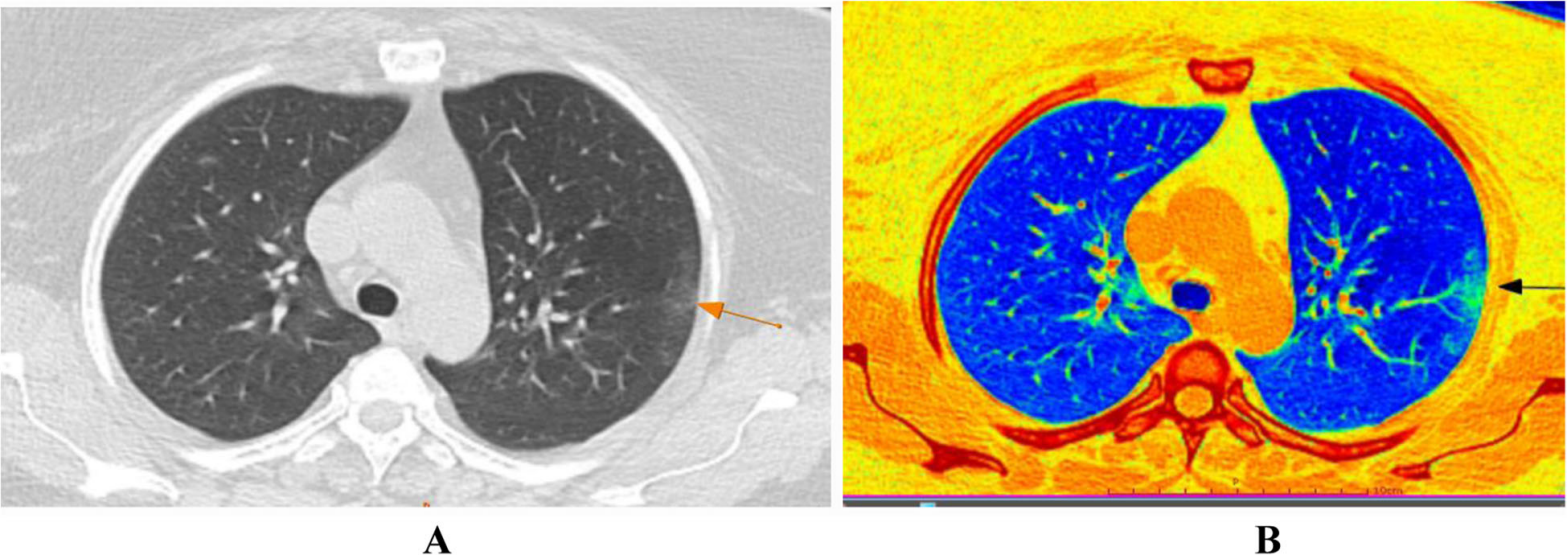
A 40-year-old female patient with first presentation with minimal cough and sore throat; CT-SSS was 2. Follow-up revealed complete resolution of the symptoms after 13 days from the onset. **a** MDCT chest, axial cuts show suspected faint peripheral sub-pleural patchy ground glass opacity seen in the left lung upper lobe apico-posterior segment; confirmed by the color coded images (**b**) with HU more than − 750

Chest CT findings were assessed and analyzed for all examined patients. Accordingly, ground glass opacities were the predominant radiological finding; pure ground glass opacities (GGO) presented in 110 patients (57.3%), mixed ground glass opacities and consolidation in 79 patients (41.1%), and pure consolidation in 3 patients (1.6%) (Table 2). Regarding disease distribution, most of the cases exhibit bilateral pulmonary affection (74%) with predominant peripheral/subpleural location (87%) (Figs. 1 and 2). Mild pleural effusion noted in 3 patients (1.6%). No pulmonary cavitations or significant lymphadenopathy was detected.

**Table 2:** Pattern of pulmonary affection in our COVID-19 pneumonic patients

Pattern	***N*** =192
GGO (ground glass opacity)	110(57.3%)
GGO and consolidation	79 (41.1%)
Consolidation	3 (1.6%)

The CT-SSS ranged from 1 to 21, with a mean value of 5.77 ±6.09. CT-SSS was significantly higher in severe cases (*P* value < 0.001).

Following recovery from the acute stage of the disease, 77 patients out of 192 developed post-COVID syndrome (40.1%).

The average age of patients who developed post-COVID syndrome was 41.05±15.15 years which was significantly higher than that of patients who did not develop post-COVID syndrome 34.20±10.50 (*P* value= 0.009).

Patients with at least one medical comorbidity (diabetes, hypertension, chronic chest, or heart diseases) were more likely to develop post-COVID syndrome (*P* value <0.05).

**Fig. 3:**
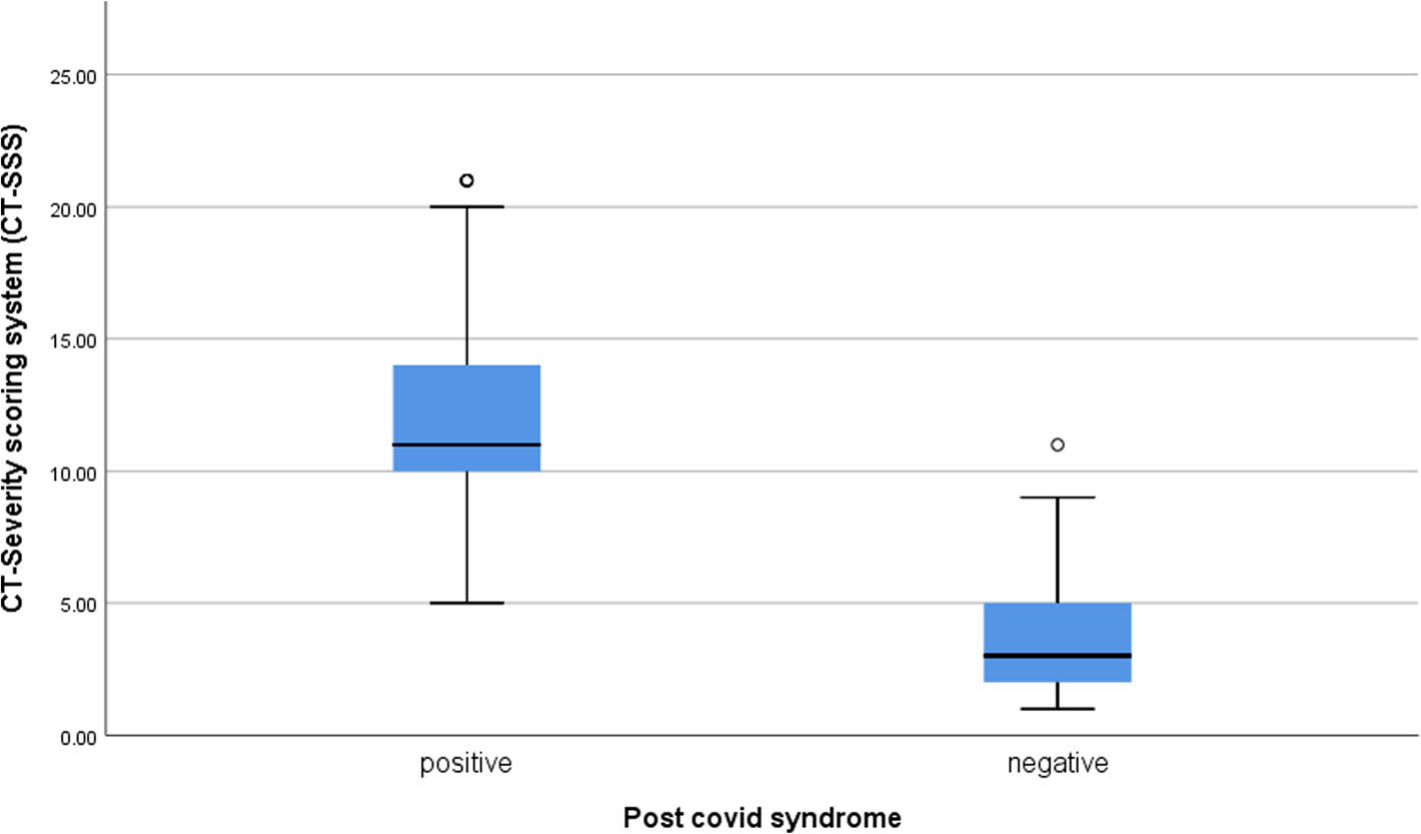
Correlation between CT-SSS and development of post-COVID syndrome

CT-SSS in patients who developed post-COVID syndrome ranged from 5 to 21 (mean 12.34±3.77) and is significantly higher than those who did not developed post-COVID syndrome (*P* value< 0.001) (Table 3, Fig. 3).

**Table 3:** Correlation between CT-SSS and development of post-COVID syndrome

	Post-COVID syndrome										
	Positive					Negative					***P*** value
	Mean	SD	Median	Minimum	Maximum	Mean	SD	Median	Minimum	Maximum	
**CT severity scoring system (CT-SSS)**	12.34	3.77	11.00	5.00	21.00	3.35	2.22	3.00	1.00	11.00	< 0.001

**Fig. 4:**
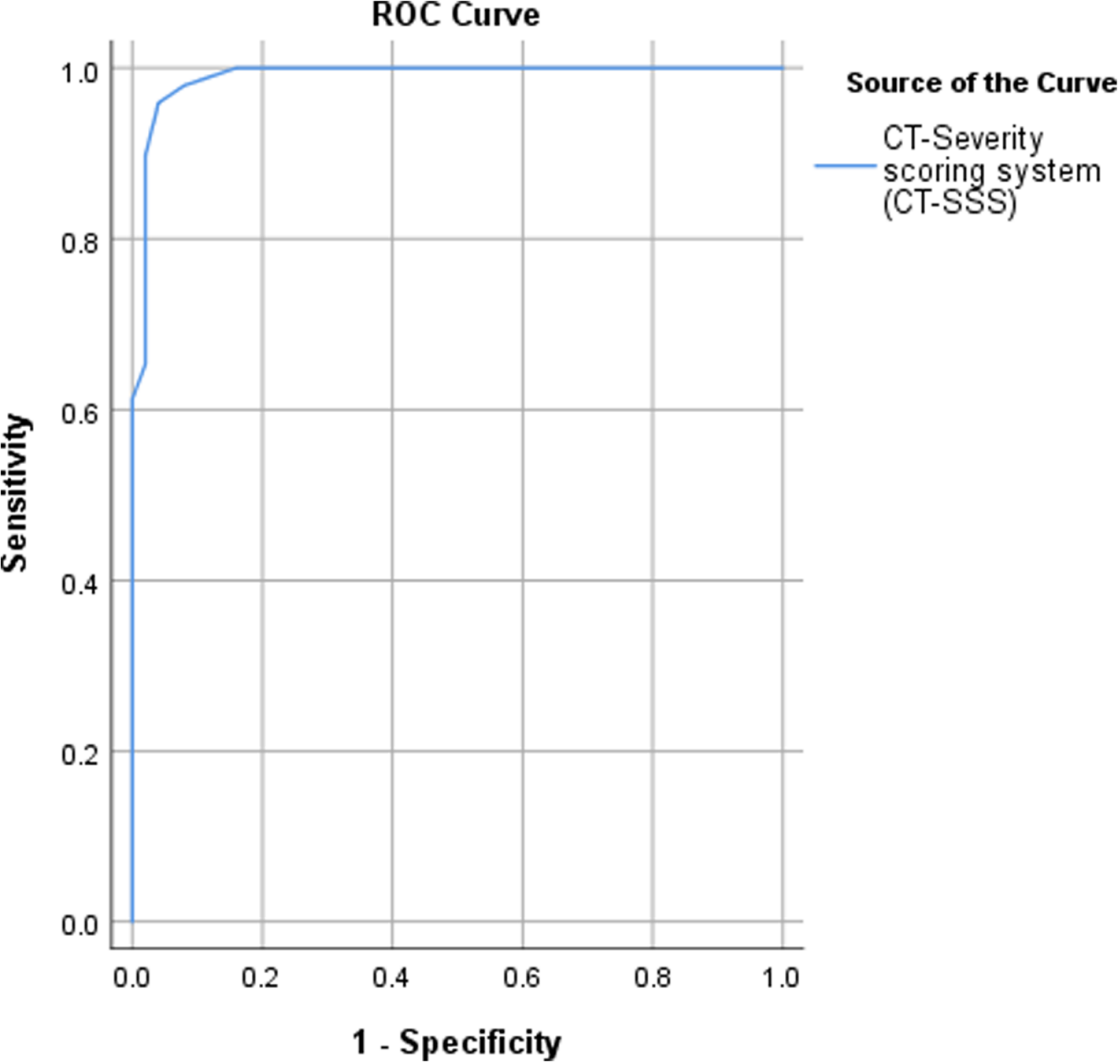
ROC curve for development of post-COVID syndrome using CT-SSS

By ROC analysis, the area under curve (AUC) was significantly high for CT-SSS with cutoff point > 7 for development of post-COVID syndrome, with sensitivity (95.9%), specificity (96%), positive predictive value (95.92%), negative predictive value (96%), and accuracy (95.96%) (Fig. 4).

From the 77 cases who developed post-COVID syndrome, only 4 cases showed a CT severity score ≤ 7. Two cases of them performed their CT at the day of admission and during the following 5 days the clinical symptoms of the patients worsen yet, as both patients were females in the reproductive period; the decision was not to expose the patients to another CT dose.

## Discussion

Since December 2019, a cluster of cases with unknown pneumonia with similar clinical manifestations suggesting viral pneumonia appeared in Wuhan city, Hubei Province, China. A new type of coronavirus was isolated from the lower respiratory tract samples, named severe acute respiratory syndrome coronavirus-2 (SARS-CoV-2) by the International Committee on Taxonomy of Viruses [1]. The disease it causes was named coronavirus disease 2019 (COVID-19) by the World Health Organization on February 11, 2020 [12].

Patient testimony is showing that a considerable number of patients would not recover totally from the effects of the virus months after their initial illness. Symptoms are wide-ranging and can include breathlessness, chronic fatigue, “brain fog”, anxiety, and stress. The NICE guideline scope published on 30 October 2020 defines post-COVID syndrome as signs and symptoms that develop during or following an infection consistent with COVID-19 which continue for more than 10–12 weeks and are not explained by an alternative diagnosis. The definition says the condition usually presents with clusters of symptoms, often overlapping, which may change over time and can affect any system within the body. It also notes that many people with post-COVID syndrome can also experience generalized pain, fatigue, persisting high temperature, and psychiatric problems [13].

CT scan can be a useful tool in evaluating the individual disease burden [14]. The severity can be assessed using a visual method (semi-quantitative, as in our study) or using a software that quantitatively determines the percentage of affected lung volumes using the deep learning algorithms [15–17].

In this study, we tried to assess the utility of the CT severity scoring system as a predictor for possible development of post-COVID syndrome in recovered patients.

From April 2020 to October 2020, 192 symptomatic COVID-19 patients were enrolled in this single-center study and high-resolution chest CT examinations were evaluated. A previously validated semi-quantitative CT score based on the lobar extent of disease as reported by Yang et al. and Pan et al. [6, 7] was calculated.

Following recovery from the acute stage of the disease, 77 patients out of 192 developed post-COVID syndrome (40.1%).

On reviewing previously published studies, the percentage of cases which developed post-COVID syndrome ranged from 32 to 60%. This wide range may be explained by the variability in study duration and number of cases [18–20].

CT-SSS in patients who developed post-COVID syndrome is significantly higher than in those who did not develop post-COVID syndrome (*P* value < 0.001).

We were able to demonstrate that a cut-off value of >7 in CT-SSS is highly predictive of long-term clinical status with a sensitivity, specificity, PPV, NPV, and accuracy of 95.9%, 96%, 95.92%, 96%, and 95.96% respectively. To our knowledge, and till the date of publication, no previous study assessed such a relationship.

We also found that either older patients or patients with at least one medical comorbidity (diabetes, hypertension, chronic chest, or heart diseases) were more likely to develop post-COVID syndrome. This could be explained by the significant correlation of those comorbidities with disease burden in COVID-19 patients and accordingly severity of lung affection and CT-severity score. This agrees with the study of Lu et al. [21] which stated that older age and increased blood glucose level were correlated with the severity of lung involvement and clinical prognosis in COVID-19 patients. There was a positive correlation between blood glucose level on admission and lung lesions.

However, our study had some limitations including limited number of patients. Furthermore, this is a hospital not a population-based study, yet, we could deduct the percentage of different CT imaging categories. It is thus recommended to perform future studies to confirm the generalizability of this study on a larger scale. We recommend also further studies to investigate other predictive parameters for development of post-COVID syndrome.

## Conclusion

CT severity scoring can help in predicting the long-term outcome of COVID-19 patients and development of post-COVID syndrome with cutoff value of CT-SSS > 7 shows highest sensitivity and specificity for predicting development of post-COVID syndrome.

## Data Availability

The datasets used and/or analyzed during the current study are available from the corresponding author on a reasonable request.

## Abbreviations

2019-COV: 2019 coronavirus
ARDS: Acute respiratory distress syndrome
CDC: Chinese center of disease control
COVID-19: Coronavirus disease
CT: Computed tomography
CT-SSS: Computed tomography severity scoring system
GGO: Ground glass opacity
kVp: Kilovoltage peak
mAs: Milliampere-seconds
MDCT: Multi-detector computed tomography
MOD: Multiple organ dysfunction
MOF: Multiple organ failure
MSCT: Multi-slice computed tomography
NICE: National institute for health and clinical excellence
NPV: Negative predictive value
PPV: Positive predictive value
ROC: Receiver operating characteristic
RT-PCR: Reverse transcriptase polymerase chain reaction
SARS-Cov-2: Severe acute respiratory syndrome coronavirus 2
SpO_2_: Blood oxygen saturation
SPSS: Statistical package for the social science
WL: Window level
WW: Window width
